# Early Tooth Loss after Periodontal Diagnosis: Development and Validation of a Clinical Decision Model

**DOI:** 10.3390/ijerph18031363

**Published:** 2021-02-02

**Authors:** Francisco Santos, Frederico Beato, Vanessa Machado, Luís Proença, José João Mendes, João Botelho

**Affiliations:** 1 Clinical Research Unit (CRU), Centro de Investigação Interdisciplinar Egas Moniz (CiiEM), Egas Moniz—Cooperativa de Ensino Superior, CRL, 2829-511 Almada, Portugal; franciscosantos.em@gmail.com (F.S.); frederico.beato@gmail.com (F.B.); vmachado@egasmoniz.edu.pt (V.M.); jmendes@egasmoniz.edu.pt (J.J.M.); 2 Department of Periodontology, CRU, CiiEM, Egas Moniz—Cooperativa de Ensino Superior, CRL, 2829-511 Almada, Portugal; 3 Evidence-Based Hub, CRU, CiiEM, Egas Moniz—Cooperativa de Ensino Superior, CRL, 2829-511 Almada, Portugal; lproenca@egasmoniz.edu.pt; 4 Quantitative Methods for Health Research (MQIS), CiiEM, Egas Moniz—Cooperativa de Ensino Superior, CRL, 2829-511 Almada, Portugal

**Keywords:** periodontal disease, periodontitis, early tooth loss, predictive model, risk factors, oral health, public health, epidemiology

## Abstract

The aim of this study was to develop and validate a predictive early tooth loss multivariable model for periodontitis patients before periodontal treatment. A total of 544 patients seeking periodontal care at the university dental hospital were enrolled in the study. Teeth extracted after periodontal diagnosis and due to periodontal reasons were recorded. Clinical and sociodemographic variables were analyzed, considering the risk of short-term tooth loss. This study followed the transparent reporting of a multivariable prediction model for individual prognosis or diagnosis (TRIPOD) guidelines for development and validation, with two cohorts considered as follows: 455 patients in the development phase and 99 in the validation phase. As a result, it was possible to compute a predictive model based on tooth type and clinical attachment loss. The model explained 25.3% of the total variability and correctly ranked 98.9% of the cases. The final reduced model area under the curve (AUC) was 0.809 (95% confidence interval (95% CI): 0.629–0.989) for the validation sample and 0.920 (95% CI: 0.891–0.950) for the development cohort. The established model presented adequate prediction potential of early tooth loss due to periodontitis. This model may have clinical and epidemiologic relevance towards the prediction of tooth loss burden.

## 1. Introduction

Periodontitis is a chronic multifactorial inflammatory disease associated with dysbiotic plaque and characterized by a progressive destruction of the tooth-supporting apparatus [1]. Periodontitis impacts the patient’s quality of life [2,3], however it can be improved after periodontal treatment [4]. From a systemic point of view, several lines of evidence associate periodontitis with several systemic diseases [5,6,7].

Mindful of the importance of teeth preservation in periodontitis patients, several factors have been debated to play a fundamental role in the fate of these teeth during every stage of periodontal care. Regarding the patient-related local factors, bleeding on probing burden, tooth type, angular defects at non-molar teeth and molar furcation are important characteristics to consider when we are planning periodontal interventions [8]. As a result, a concept of critical probing depth for clinical decision making was developed [9]. On the other hand, there are many systemic risk factors for periodontal disease such as diabetes mellitus [10], age [11], smoking [12] and overweight and obesity [13]. 

Each tooth is given a prognosis in order to choose the right treatment with the greatest probability of success [14]. A range of multivariable models for assessing the association between independent variables and tooth loss in periodontitis patients have been developed [15,16,17,18]. Furthermore, the available systematic evidence points to a number of influencing variables during active periodontal treatment (APT) and supportive periodontal therapy (SPT) [19], though the most striking variables before any treatment seem to rely on clinical characteristics such as the probing depth (PD), clinical attachment loss (CAL) or mobility [8,9,20]. In addition, the accuracy of most multivariable tooth loss prediction models was limited [15] and, as far as we know, there are no predicting models for early tooth loss after periodontitis diagnosis and before commencing APT.

The aim of this study was to develop and validate a predictive early tooth loss multivariable model in a sample of periodontitis patients before APT.

## 2. Materials and Methods

This study was approved by the Egas Moniz Ethics Committee (Approval numbers 733 and 818) and was carried out in accordance with the Helsinki Declaration of 1975 as revised in 2013. Written informed consents were obtained from all participants during the first appointment at Egas Moniz Dental Clinic (EMDC). Data were registered on an approved and monitored database. Patients were referred to receive appropriate treatment according to the periodontal diagnosed conditions.

This investigation follows the transparent reporting of a multivariable prediction model for individual prognosis or diagnosis (TRIPOD) reporting guidelines [21] for development and validation of prediction models (Supplementary Material Table S1). This study was conducted on a triple blinded basis with respect to diagnosis and clinical outcome, data collection and analysis.

### 2.1. Source of Data, Participants, Sample Size and Missing Data

#### 2.1.1. Source of Data

This retrospective cohort study included patients who attended the Periodontology Department of Egas Moniz Dental Clinic (EMDC) between May 2015 and March 2020. Data were sourced from a digital clinical registry database. 

#### 2.1.2. Participants and Setting

All participants were patients at EMDC, a university dental hospital, located in the municipality of Almada, in the Setúbal Peninsula (a Nomenclatura das Unidades Territoriais [NUTS] III subregion, part of NUTS II Lisbon Region), that provides dental health services to the general public.

Patients presented at the Periodontology Unit to seek periodontal care according to their Periodontal Screening Record [22] index in a triage consult. After periodontal diagnosis, teeth considered for extraction were registered and were surgically removed.

The inclusion criteria were: adult patients (18 years or older), a full-mouth complete periodontal diagnosis and signed written informed consent. The exclusion criteria were: patients with no follow-up after the periodontal diagnosis, missing data and periodontally healthy according to American Academy of Periodontology (AAP)/European Federation of Periodontology (EFP) case definition [23].

#### 2.1.3. Sample Size and Missing Data

As this study aimed to develop and validate a predictive model, the overall sample was randomly split in two, according to a temporal validation strategy [21,24]. The specific time point for the data set separation was randomly determined: 31 May 2018. Therefore, two samples emerged: development cohort, from 1 March 2015 to 31 May 2018 and validation cohort, from 1 June 2018 until 10 March 2020 (given the lockdown restrictions of the Portuguese government).

Incomplete periodontal diagnosis data led to the exclusion of the respective patient from the sample, thus no imputation methods were required.

### 2.2. Outcome

Tooth extraction assessment was performed through careful evaluation of the clinical file. Only teeth extracted due to periodontal reasons and before the beginning of the non-surgical periodontal treatment were included. Teeth extracted due to non-periodontal causes were excluded from the analysis.

### 2.3. Predictors

#### 2.3.1. Periodontal Diagnosis and Measurement Reproducibility

Five calibrated periodontists performed the examinations. Prior to the initiation of the study, all examiners had previous theoretical and practical training in a total of ten volunteer non-study patients suffering from moderate to severe periodontitis. The inter-examiner correlation coefficients, at subject level, ranged from 0.76 to 0.97 and between 0.91 and 0.99, for mean PD and mean CAL, respectively as previously reported [25].

Full-mouth periodontal examinations were performed, excluding third molars, retained roots and implants. Periodontitis and severity stages were defined according to EFP/AAP case definition [23]. The evaluated parameters were: missing teeth, PD, gingival recession and CAL. At six sites per tooth (mesiobuccal, mid-buccal, distobuccal, mesiolingual, mid-lingual and distolingual), PD was measured as the distance from the cementoenamel junction (CEJ) to the bottom of the pocket and recession as the distance from the CEJ to the free gingival margin, and this assessment was assigned a negative sign if the gingival margin was located coronally to the CEJ. CAL was calculated as the algebraic sum of PD and recession. A CP-12 SE was used during periodontal examination (Hu-Friedy, Chicago, IL, USA). Furcation involvement (FI) was assessed using a Nabers probe (2N Hu-Friedy, Chicago, IL, USA) in molars, and upper first premolars if applicable [26], and tooth mobility was appraised [27]. In addition, teeth were also categorized according to the quadrant, sextant and type (molar, premolar, canine and incisor).

#### 2.3.2. Sociodemographic Variables 

Sociodemographic variables and several periodontal disease risk factors were selected as covariates. The selected sociodemographic variables were: age, gender and smoking status. Smoking status was dichotomously defined as non-smoker or smoker. 

### 2.4. Data Collection

Data used were collected with a blinded procedure, i.e., without the examiner (who collected the data) knowing the identification of the patients. To this end, the patient list was coded and the data submitted through an online platform (Google Forms^®^, Menlo Park, CA, USA). Data were obtained from EMDC clinical record digital platform (Egas Moniz Adult Oral Health Database).

### 2.5. Statistical Analysis Methods

Data analysis was performed using IBM SPSS Statistics version 26.0 for Windows (IBM Corporation, Armonk, NY, USA). Descriptive and inferential statistics methodologies were applied. In the latter, Mann–Whitney and Chi-squared tests were used to compare the clinical data as a function of the cohort type and categories/groups, as identified by clinical and sociodemographic variables. Further, logistic regression analysis was used to model the relationship between the clinical outcome (surgery) and several risk indicators. Preliminary analyses were performed using univariate models. Next, a multivariate model was constructed for the outcome variable (surgery). Only variables showing a significance *p* ≤ 0.25 in the univariate model were included in the multivariate stepwise procedure. Predictor variables considered in this procedure were: smoking status, tooth type, PD, recession and CAL. The contribution of each variable to the model was evaluated by Wald statistics. Interactions were also analyzed for all tested variables. The final reduced model was obtained with the following predictor variables: tooth type (incisor or not) and CAL. Odds ratio (OR) and 95% confidence intervals (95% CI) were calculated for both univariate and multivariate analyses. The level of statistical significance was set at 5%.

## 3. Results

**Figure 1:**
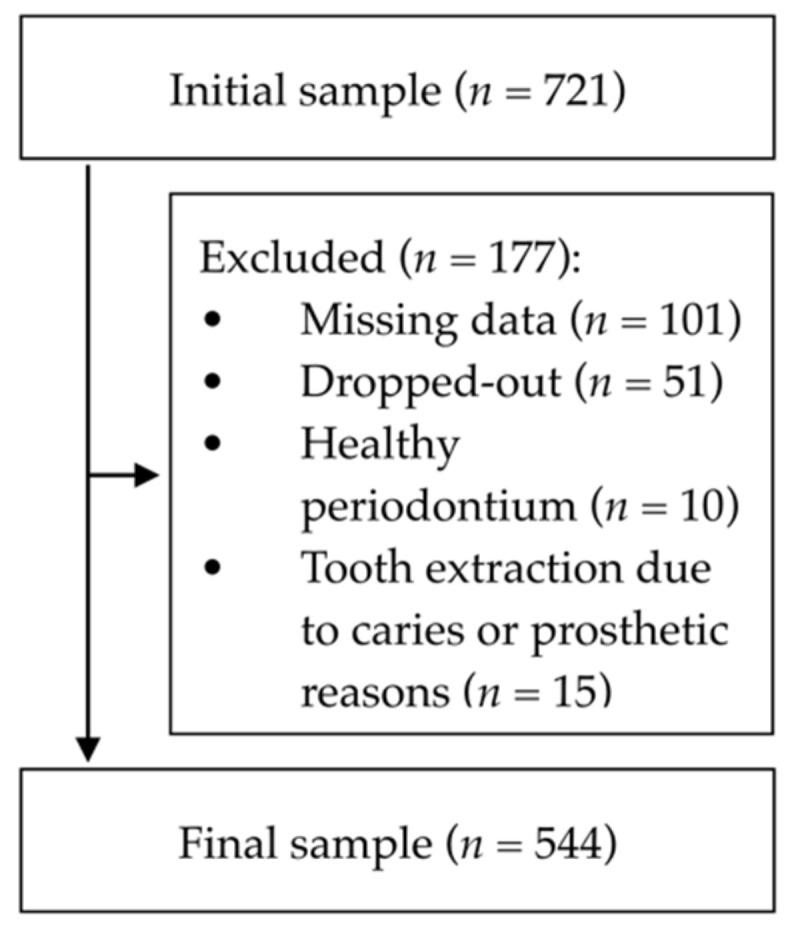
Participants flowchart. An initial sample of 721 participants were consecutively enrolled, with a final sample of 544 participants included in the study, after confirming the inclusion criteria.

From an initial sample of 721 patients, 544 (75.5%) (aged 18 to 90 years old) were considered for this study (Figure 1), with 101 individuals excluded due to missing data, 51 patients dropped out, 15 were excluded since their teeth extractions were due to non-periodontal causes, specifically for prosthetic reasons and irreparable caries, and 10 patients were considered periodontally healthy according to the current EFP/AAP classification.

According to the defined time point of separation, 455 patients were eligible for the model development, while the remaining 99 individuals were used for validation purposes. Table 1 presents the characteristics of the included participants.

Comparing both the development and validation cohorts, only one variable (age) exhibited significant differences (*p* < 0.001). When considering gender, stage of periodontitis and smoking status, no significant differences were observed (*p* > 0.05). This was also the case for the number of missing teeth, teeth with mobility, teeth extracted and for the periodontal clinical variables PD, CAL and recession (*p* > 0.05). Thus, both samples showed similarities and balanced distribution accordingly. Furthermore, the proportion of teeth extracted after periodontal diagnosis was not significantly different between both samples (*p* = 0.057).

When considering the association between potential confounding variables and tooth extraction (Table 2) it can be seen that smoking status and tooth type exhibited an association with the clinical outcome variable (*p* = 0.006 and *p* < 0.001, respectively).

In the model development cohort, extracted teeth had an average PD of 4.99 mm (SD = 1.43), recession of 2.02 mm (SD = 1.02), CAL of 7.01 mm (SD = 2.26) and 7.38 (SD = 4.84) missing teeth (Table 3). The teeth that were not indicated for surgery had a mean PD of 2.97 mm (SD = 1.08), recession of 0.71 mm (SD = 1.2), CAL of 3.67 mm (SD = 1.59) and 5.16 (SD = 4.59) missing teeth. In all cases significant differences were observed between the two groups (*p* < 0.001).

When considering the development cohort, the crude model presented several important factors to be explored in a multivariate approach (Table 4). The categories active ‘smoker’ (OR = 0.58, 95% CI: 0.39-0.86, *p* = 0.007), ‘tooth type (incisor)’ (OR = 2.10, 95% CI: 1.25-3.54, *p* = 0.005) and the clinical variables PD (OR = 2.92, 95% CI: 2.55-3.34, *p* < 0.001), recession (OR = 1.94, 95% CI: 1.76-2.15, *p* < 0.001) and CAL (OR = 1.88, 95% CI: 1.75–2.03, *p* < 0.001), were identified as potential factors to be considered.

Based on the results obtained in the evaluation by univariate models, a multivariate logistic regression model for tooth loss was created (Table 4). In this multivariable model only the factors ‘tooth type (incisor)’ and CAL were identified as significant predictors (*p* = 0.037 and *p* < 0.001, respectively). In the final reduced model, the equation coefficient (B) values for incisor and CAL were determined as 0.589 and 0.661, respectively. Factor risks were quantified by the correspondent OR, with values of 1.80 (95% CI: 1.04–3.12) for incisor and 1.94 (95% CI: 1.78–2.10) for CAL.

Thus, based on these values, an equation modeling tooth loss prediction (extraction by surgery), in periodontitis patients, could be identified as:

log [Probability (extraction)/(1 − Probability (extraction))] = −7.850 + (0.589 × incisor) + (0.661 × CAL)(1)

where the variable category ‘incisor’ is codified as ‘1’ if the tooth extracted is an incisor. The CAL value refers to the mean value of a circumferential assessment with six measurements.

**Figure 2:**
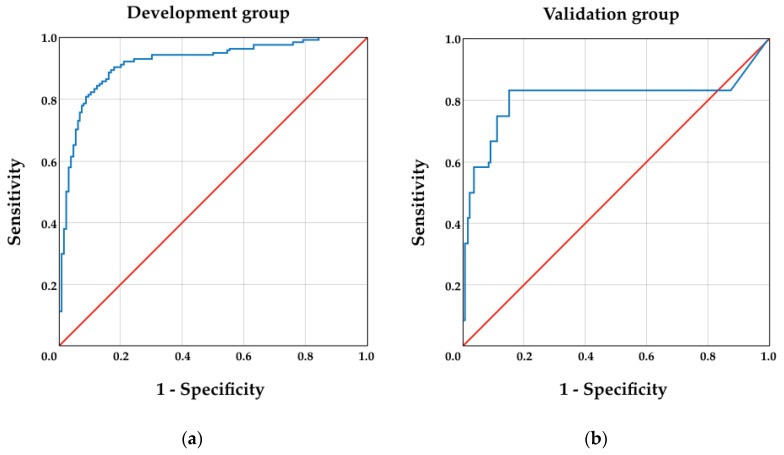
Receiver-operating characteristic curves (ROC) for the development (**a**) and validation (**b**) cohorts.

The obtained area under the curve (AUC) with the final reduced model was 0.809 (95% CI: 0.629–0.989) for the validation sample and 0.920 (95% CI: 0.891–0.950) for the development cohort, as illustrated in Table 5. The receiver-operating characteristic (ROC) curves of both groups are graphically shown in Figure 2.

## 4. Discussion

The aim of this study was to develop and validate a prediction model of early tooth loss in patients diagnosed with periodontitis at a reference dental hospital. Overall, we were able to develop and validate a model with high sensitivity and specificity characteristics. This simple model accounted for periodontal clinical characteristics and may have noteworthy potential for both clinical and epidemiologic purposes.

Comprehensively, tooth extraction due to periodontal reasons immediately after periodontal diagnosis was seen as a rare event, considering that 1.1% and 0.6% of teeth were extracted in the development and validation samples, respectively. One reason for this event is the university setting of Clínica Dentária Egas Moniz (CDEM), even though there are reported differences to practice-based scenarios [19]. In addition, this university clinic employs a minimally invasive dentistry methodology that has beneficial clinical effects [28]. Together, this model was initially designed for an individual dental prognosis, so it should be borne in mind that the fate of a tooth is often influenced by the final overall treatment plan that involves the entire dentition [16].

Nevertheless, the epidemiologic potential is worth mentioning. This model accounts for solely clinical measures and with high reliability performance, hence it may be commissioned in large-based studies to predict tooth loss in the short-term. To the public health view, this conceivable information might be crucial to delineate and prepare appropriate measures to target tooth loss and edentulism, both recognized as disturbing public health problems and with devastating consequences for oral health-related quality of life [2,29,30].

Although the risk result associated with smoking habits has a controversial expression, this was not included in the final multivariable model, since, in its elaboration, it was not statistically significant. Both PD and recession are not present in the multivariable model, and the main reason might reside in the fact that both are used to compute CAL, and for this reason collinearity was circumvented.

The remaining significant variable in the final predictive model was the type of tooth, specifically being an incisor. This result is in agreement with literature, in which incisors have higher risk of to be lost [8,9], due to less bone anchorage, contrary to canines (also monoradicular teeth) that have longer roots and are more prevalent in the oral cavity of the CDEM population [25].

Beyond the advantages of assisting the clinician in deciding the prognosis of a tooth and the best treatment option [31], predictive risk models may have positive consequences as they provide a risk assessment and early future treatment [32], taking a preventive approach to improve oral health-related quality of life.

This study presents important limitations that preclude wide generalization. As a retrospective study, this investigation has controls by convenience sampling, temporal relationships are difficult to infer. Nevertheless, the high sample and the employment of up-to-date case definitions have certainly contributed to minimize these biases. Moreover, we were not able to further deepen our knowledge on systemic conditions, for example diabetic or hypertension. Despite the availability of self-reported diabetes and high blood pressure, we found it unreasonable to include them into the analysis as this would contribute to biasing the analysis since we lack clinically relevant data (i.e., glycated hemoglobin, relevant medication, etc.). Yet, the extensive clinical assessment allowed for a holistic view and a model that explains 25.3% of variability and with high accuracy performance. Considering that tooth extraction was a rare event, larger samples would be desirable, although the proposed model demonstrated high consistency. In addition, this study developed and validated the prediction model in a separate sample and this might be seen as an advantage [33], however in the future this model shall be investigated in wider and regional- or national-based samples.

## 5. Conclusions

The developed predictive model of early tooth loss due to periodontitis, significantly depends on tooth type and clinical attachment loss. During validation, this model presented adequate prediction potential for early tooth loss. The developed model may have clinical and epidemiologic relevance towards the prediction of tooth loss burden.

## Figures and Tables

**Table 1:** Characteristics of the development and validation cohorts.

Variable	Development Cohort (*n* = 455)	Validation Cohort (*n* = 99)	*p*-Value ^1^
Gender, *n* (%)			
Male	248 (54.5)	56 (56.6)	0.709
Female	207 (46.5)	43 (43.4)	
Age (years), mean (SD)	54.8 (12.5)	45.8 (16.3)	<0.001
Periodontitis, *n* (%)			
Stage I (Mild)	16 (3.5)	5 (5.1)	0.378
Stage II (Moderate)	140 (30.8)	36 (36.4)	
Stage III/IV (Severe/Advanced)	299 (65.7)	58 (58.6)	
Smoking status, *n* (%)			
Smoker	241 (53.0)	44 (44.4)	0.124
Non-smoker	214 (47.0)	55 (55.6)	
Teeth with mobility (*n*), mean (SD)	0.2 (0.5)	0.1 (0.3)	0.467
Missing teeth (n), mean (SD)	6.8 (5.6)	5.6 (5.5)	0.078
PD (mm), mean (SD)	2.3 (1.6)	2.2 (1.5)	0.218
Recession (mm), mean (SD)	0.6 (1.0)	0.4 (0.8)	0.167
CAL (mm), mean (SD)	2.8 (2.1)	2.3 (1.6)	0.109
Teeth extracted, *n* (% from total)	103 (1.1)	12 (0.6)	0.057

CAL—clinical attachment loss; PD—probing depth; SD—standard deviation. ^1^ Mann–Whitney and Chi-squared tests, for continuous and categorical variables, respectively.

**Table 2:** Association between tooth extraction and confounding variables in the development cohort.

Confounder	*p*-Value ^1^
Gender	0.115
Smoking status	0.006
Quadrant	0.360
Sextant	0.636
Tooth type	<0.001
Mobility	NA
Furcation	NA

^1^ Chi-squared test. NA—not applicable.

**Table 3:** Comparison of periodontal characteristics of teeth, as a function of the clinical outcome.

Variable	Surgery		*p*-Value ^1^
	Yes	No	
PD (mm), mean (SD)	4.99 (1.43)	2.97 (1.08)	<0.001
Recession (mm), mean (SD)	2.02 (1.72)	0.71 (1.20)	<0.001
CAL (mm), mean (SD)	7.01 (2.26)	3.67 (1.59)	<0.001
Missing Teeth (n), mean (SD)	7.38 (4.84)	5.16 (4.59)	<0.001

CAL—clinical attachment loss; PD—probing depth; SD—standard deviation. ^1^ Mann–Whitney test.

**Table 4:** Crude and adjusted logistic regression models towards tooth extraction after periodontal diagnosis (*n* = 455).

Variable	OR (95% CI)	*p*-Value	OR (95% CI)	B ^1^	*p*-Value
Smoking					
Non-smoker	1	-	-	-	-
Smoker	0.58 (0.39–0.86)	0.007	-	-	-
Tooth Type					
Incisor	2.10 (1.25–3.54)	0.005	1.80 (1.04–3.12)	0.589	0.037
Canine	0.46 (0.19–1.18)	0.108	-	-	-
Premolar	1	-	-	-	-
Molar	1.70 (0.95–3.04)	0.073	-	-	-
PD (mm)	2.92 (2.55–3.34)	<0.001	-	-	-
Recession (mm)	1.94 (1.76–2.15)	<0.001	-	-	-
CAL (mm)	1.88 (1.75–2.03)	<0.001	1.94 (1.78–2.10)	0.661	<0.001

95% CI—95% confidence interval; CAL—clinical attachment loss; PD—probing depth; OR—odds ratio ^1^ B—equation coefficient. The reduced model explains 25.3% of total variability and correctly ranks 98.9% of the cases.

**Table 5:** Receiver-operating characteristic curve analysis for both the development (*n* = 455) and validation samples (*n* = 99).

Group	AUC	95% CI	
		Lower Limit	Upper Limit
Development	0.920	0.891	0.950
Validation	0.809	0.629	0.989

95% CI—95% confidence interval; AUC—area under the curve.

## Data Availability

Data available upon reasonable request.

## Supplementary Materials

The following are available online at https://www.mdpi.com/1660-4601/18/3/1363/s1, Table S1: Tripod Checklist: Prediction Model Development and Validation.
